# The complete chloroplast genome of *Sisymbrium irio*

**DOI:** 10.1080/23802359.2018.1464412

**Published:** 2018-04-23

**Authors:** Takahiro Kawanabe, Hiroaki Nukii, Hazuka Y. Furihata, Takanori Yoshida, Akira Kawabe

**Affiliations:** Faculty of Life Science, Kyoto Sangyo University, Kyoto, Japan

**Keywords:** *Sisymbrium*, chloroplast, Brassicaceae

## Abstract

The complete chloroplast genome of *Sisymbrium irio* was determined. The length of the complete chloroplast genome is 154,001 bp. The whole chloroplast genome consists of 83,891 bp long single copy (LSC) and 17,630 bp small single copy (SSC) regions, separated by a pair of 26,240 bp inverted repeat (IR) regions. The *S. irio* chloroplast genome encodes 112 annotated known unique genes including 79 protein-coding genes, 30 tRNA genes, and four rRNA genes. The phylogenetic position of *S. irio* is sister to Brassiceae and Thlaspideae.

*Sisymbrium irio* belongs to the tribe Sisymbrieae in the family Brassicaceae. The tribe Sisymbrieae is phylogenetically close to the tribe Brassiceae, included in the Lineage II (Bailey et al. 2006; German et al. 2009; Beilstein et al. 2010; Couvreur et al. 2010; Warwick et al. 2010; Huang et al. 2016). *Sisymbrium irio*, known as London Rocket, is a common weed and used for analyses of repetitive sequences and comparative studies of tribe Brassiceae (Grellet et al. 1989; Hall et al. 2011). Due to its production of a large number of seeds, this species had been naturalized worldwide as invasive species and are found often abundant. Draft genome sequence of *S. irio* was already determined for comparative genomic analysis (Haudry et al. 2013).

A strain sampled from Pakistan (ABRC accession CS22563, SASSC J04) was used for sequencing. Chloroplast was isolated as described in Okegawa and Motohashi (2015). DNAs were extracted from the isolated chloroplasts by DNeasy Plant Mini kit (Qiagen, Hilden, Germany) and sequenced as the single-ended reads using the NextSeq500 platform (Illumina Co., San Diego, CA). The generated reads were assembled by velvet 1.2.10 (Zerbino and Birney 2008) and were also mapped to *Sinapis arvensis* whole chloroplast genome sequence (GenBank accession # KU050690) using bowtie (Langmead and Salzberg 2012) to construct complete chloroplast genome sequence. The average depth of chloroplast genome was 845.7. The nucleotide sequence was submitted to DDBJ (accession number: LC375846).

The complete chloroplast genome of *S. irio* has a total length of 154,001 bp, consisting of 83,891 bp LSC region and 17,630 bp SSC region separated by a pair of 26,240 bp inverted repeat (IR) regions. This structure is identical to those of other species in Brassicaceae. The overall GC content is 36.32% and the GC contents of the LSC, SSC, and IR regions are 34.09%, 29.24%, and 42.26%, respectively. There are 86 protein coding genes, 37 tRNA genes, and eight rRNA genes typical for the chloroplast genome of Brassicaceae. Most genes occur as a single copy, except for seven protein coding, seven tRNA, and four rRNA genes within the IR regions are duplicated in whole chloroplast genome. Sometimes, a few chloroplast genes were lost in many plant species (Guo et al. 2017), however, *S. irio* has complete set of protein coding genes in Brassicaceae species without any pseudogenization.

The phylogenetic tree including *S. irio* and species in Lineage II and EII was constructed by complete chloroplast genomes (Figure 1). 76 protein coding gene sequences were concatenated to estimate NJ tree with JC corrected synonymous divergence by MEGA ver. 7 (Kumar et al. 2016). *Sisymbrium irio* was sister to a clade containing Brassiceae and Thlaspideae species as reported by phylogenetic studies with partial chloroplast and nuclear sequences (Beilstein et al. 2010; Couvreur et al. 2010), whereas nuclear ITS regions showed different clustering where Sisymbrieae clustered with Isatideae and Schizopetaleae (German et al. 2009; Warwick et al. 2010). The complete chloroplast genome sequence of *S. irio* can provide a reference for other Sisymbrium species including many invasive weeds.

**Figure 1.**
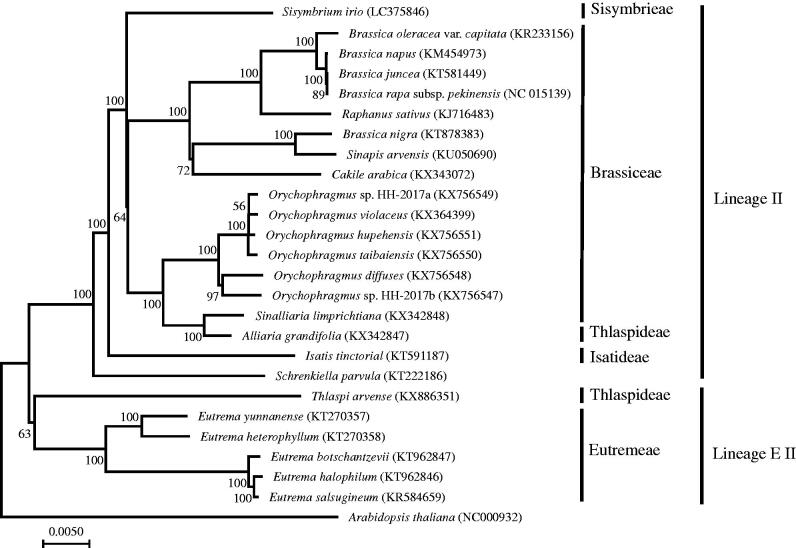
Phylogenetic of Lineage II and EII in the family Brassicaceae based on the neighbour-joining analysis of the concatenated CDSs from whole chloroplast genome with 500 bootstrap replicates.
